# The Veteran Commodore

**Published:** 1873-07

**Authors:** 


HALL'S

### JOURNAL OF HEALTH.

VOL. XX.—JULY, 1873.—NO. VII.

### THE VETERAN COMMODORE.

THERE was a man, many years ago, who worked hard, dressed plainly, lived almost meanly economical; was never known to have helped a human being to the smallest amount of current coin; nobody liked him, and yet nobody could say anything against him, for he was a peaceable citizen, was just and fair in all his dealings, and was always as good as his word. Still people did not like him; the richer he grew the more fierce they were in applying the epithet "Mean." Sixty years earlier, when the son of a very poor widow, he had often witnessed the painful labor it occasioned his mother to get water enough for the wants of the family, as it had to be brought from a great distance by hand.

When he died he left all his fortune to be appropriated towards conveying water into the town, to be free to all who wanted to use it. Everybody was ashamed of their misjudgment, and his name and memory is "blessed" to this day. He formed an unalterable purpose in childhood to supply the poor of the place with abundant water—bent all his energies to that one purpose, made its accomplishment the work of his life—and he succeeded.

Our own noble Peter Cooper, from the hardship of getting a little learning while he was a child, because he had no books to learn from, had the wish and cherished the hope that he might be able some day to do something to aid boys in having access to what books they needed, in the acquisition of knowledge enough to make their way in the world, and to that end he has given nearly a million of dollars, and everybody to-day hears the mention of his name with affection and respect. And now another man has taken the whole country by surprise and given half a million of dollars to found a Methodist University. The donation has been so kingly, and so wholly unexpected, that every one has been completely taken aback; the people don't know what to think of it, and are asking how did it come about? This was the way of it:

THERE WAS A WOMAN IN THE CASE.

A few months ago a Bishop of the Methodist Episcopal Church in the South had occasion to consult a New York physician upon a form of disease which would require his remaining in the city for some weeks, accordingly he engaged rooms at a New York hotel. But that good man, that wide-awake and large hearted worker in the Master's vineyard, Rev. Dr. Deems, heard of it, and very soon a lady heard of it, and in double quick time, according to orders, no doubt, the old Commodore called on the Bishop and said to him, "Bishop, it will never do for you to stay at a hotel; you can't get the attention here which you will require; you must come to my house."

"My dear sir," replied the Bishop, "I couldn't think of burdening your family with the care of me for so long a time, and a hotel is a much more suitable place than a private family."

"Well, Bishop, my house is a hotel—come along;" and off they went to the princely mansion on Washington Square, where, under the hands of that able and eminent surgeon, Bodenhamer, the good man was restored to perfect health, after having been a great sufferer for weary years, and had completely failed of relief under the hands of the most skilful physicians of the country, North and South.

Meanwhile Dr. Deems, the Bishop's old friend, made frequent calls, to encourage and to cheer, but every time they met their conversation drifted to the subject of establishing a denominational University in the South, considering it was an imperative necessity fort he extension of their lines and the public good. Oftentimes the old Commodore was present, and now and then seemed to be an attentive listener, but said nothing. One day, however, he said to the Bishop, when they were alone, "Bishop, tell me something about this college of yours, in which you and Dr. Deems seem to take such an interest." The needed information was given, concluding, however, with the statement that the accomplishment of the plan was in the far future, as the means of the people in that section of the country were very limited. In his off hand way the Commodore said he would give him five hundred thousand dollars toward the object. The announcement came near being the death of the good Bishop; he couldn't exactly make out whether he was standing on his head or heels. "I don't know how I'll ever get home." His feeling was that the news was so good, and would be hailed with such rapture by his brethren, and his own heart was so full of gratitude, that it was rather a doubt whether or not he might not explode entirely.

Some few years ago the Commodore married a young lady from the South, a distant connection, it was said, and when it is further stated that she was

A GOOD METHODIST, the results will not be wondered at. She is a noble hearted woman. Her head was never turned by becoming the wife of a man worth fifty millions, so said; amid all the surroundings of enormous wealth she has never forgotten the God of her fathers, nor the Church of her youth, and from the mere force of Christian principle and duty she has, from the first, so deported herself as to make herself a power for good, and who knows but what, like another Esther, she has been raised up for this very thing, and that it is through her Christian and wifely bearing her husband gave first a church to her people and then a university to her denomination—a university which shall be educating the sons of the Church and sending them into the ministry by dozens and by scores every year, in the not far distant future, to bear the tidings of the Gospel from Knoxville, as a new Jerusalem, spreading out all over this great land of ours, and extending, and extending, until there shall be

"A CIRCUIT RIDER"

in every State and Territory in the nation, and a Methodist missionary among every tribe, and tongue, and people, through the instrumentality of

CORNELIUS VANDERBILT, the man who once made a living by rowing people across the Hudson River, between New York and Jersey City, for twelve and a half cents apiece. Tell me, my reader, is this not more worth living for than to have founded the Pyramids—than to have ruled an empire—an accomplishment greater than that of Alexander, and Cæsar, and Napoleon—

greater than all combined? And now let me suggest one little thing—that the Christian men and women of every name and sect "pray without ceasing" that the generous donor, now near fourscore, may not fail of an entrance into eternal life at last; but be diligent herein, "for the time is short."

